# Sex-Biased CHHs and Their Putative Receptor Regulate the Expression of IAG Gene in the Shrimp *Litopenaeus vannamei*

**DOI:** 10.3389/fphys.2019.01525

**Published:** 2019-12-20

**Authors:** Qing Guo, Shihao Li, Xinjia Lv, Jianhai Xiang, Rivka Manor, Amir Sagi, Fuhua Li

**Affiliations:** ^1^Key Laboratory of Experimental Marine Biology, Institute of Oceanology, Chinese Academy of Sciences, Qingdao, China; ^2^Laboratory for Marine Biology and Biotechnology, Qingdao National Laboratory for Marine Science and Technology, Qingdao, China; ^3^Center for Ocean Mega-Science, Chinese Academy of Sciences, Qingdao, China; ^4^University of Chinese Academy of Sciences, Beijing, China; ^5^Department of Life Sciences and the National Institute for Biotechnology in the Negev, Ben-Gurion University of the Negev, Beersheba, Israel

**Keywords:** CHH, “eyestalk-AG-testis” endocrine axis, guanylate cyclase, IAG, male sexual differentiation

## Abstract

The “eyestalk-androgenic gland (AG)-testis” endocrine axis is involved in male sexual differentiation of crustaceans. The insulin-like androgenic gland hormone (IAG), secreted from the AG, plays a central role in this axis, however key factors upstream the IAG are still poorly understood. Here, two crustacean hyperglycemic hormone (CHH) genes (*LvCHH1* and *LvCHH2*) and their putative receptor guanylate cyclase (*LvGC*) were identified in *Litopenaeus vannamei*. LvCHH1 and LvCHH2 belonged to CHH subfamily I members and LvGC was a membrane-bound guanylate cyclase. They were all differentially expressed in eyestalks and gonads of males and females. RNA interference (RNAi) of either *LvCHH1* or *LvCHH2* increased *LvIAG* expression, while injection of their recombinant protein decreased *LvIAG* expression, indicating that LvCHH1 and LvCHH2 are inhibitory factors of *LvIAG* expression. Yeast two-hybrid assay showed that both LvCHH1 and LvCHH2 interacted with LvGC and their RNAi and recombinant protein injection exerted opposite regulatory effects on the transcriptional expression of *LvGC*. Meanwhile, knockdown of *LvGC* increased *LvIAG* expression. These results suggest that LvGC is the receptor of LvCHH1 and LvCHH2 and they are all involved in male sexual development by regulating *LvIAG* expression. The present study unveils missing upstream elements in the “eyestalk-AG-testis” endocrine axis in crustacean.

## Introduction

In crustacean, sex determination and sexual differentiation, which lead to sexual dimorphism, attract an increasing attention. The androgenic gland (AG) unique to male crustaceans was first discovered in the blue crab *Callinectes sapidus* (Cronin, 1947). The function of the AG in regulating the male sexual differentiation has been confirmed in decapods such as *Macrobrachium rosenbergii* in which bilateral AG ablation led to the atrophy of the sperm ducts and testes (Sagi et al., 1990). In isopods, oogenesis was observed instead of spermatogenesis after removing the AG from male individuals, while gonadal masculinization and sex reversal were induced after AG implantation into female individuals (Suzuki and Yamasaki, 1997). The AG function was firstly identified in isopods to be mediated by a protein belonging to the insulin superfamily (Okuno et al., 1997, 1999). In decapods, insulin-like androgenic gland hormone (IAG) was firstly reported in *Cherax quadricarinatus* (Manor et al., 2007), and then it was reported in many decapod crustaceans (Banzai et al., 2011; Chung et al., 2011; Li et al., 2012; Lawrence et al., 2017) including *Litopenaeus vannamei* (*LvIAG*) (Vázquez-Islas et al., 2014). Knockdown of *IAG* by double-stranded RNA (dsRNA) mediated RNA interference (RNAi) could disrupt testis development and spermatogenesis in decapods (Ventura et al., 2009; Rosen et al., 2010), confirming that IAG secreted from the AG controls male sexual differentiation of crustaceans.

Eyestalk ablation leads to hypertrophy of the AG, change of its cell types and increase of RNA synthesis in AG cells of *Pandalus platyceros* (Foulks and Hoffman, 1974), *C. quadricarinatus* (Khalaila et al., 2002), and *Portunus pelagicus* (Sroyraya et al., 2010). In *C. sapidus* and *Fenneropenaeus chinensis*, eyestalk ablation significantly up-regulated the expression level of *IAG* (Chung et al., 2011) suggesting the existence of an “eyestalk-AG-testis” endocrine axis proposed to be involved in sexual differentiation of male crustaceans (Khalaila et al., 2002). As a major neuroendocrine structure specific to crustaceans, the X-organ/sinus-gland (XO-SG) complex located in crustacean eyestalk synthesizes and secretes neuropeptides that regulate various physiological processes, including regulation of carbohydrate metabolism, molting, growth, and reproduction (Santos and Keller, 1993; de Kleijn et al., 1994; Chung et al., 2010; Chang and Mykles, 2011). These functions are mainly performed by crustacean hyperglycemic hormones (CHHs), which are a large family of crustacean neurohormones synthesized in the medulla terminalis of the XO-SG (Lacombe et al., 1999).

Based on the preprohormone structure, CHH family neuropeptides can be divided into the subfamily I peptides containing a CHH precursor related peptide (CPRP) and the subfamily II peptides lack the CPRP (Böcking et al., 2002). The subfamily I peptides have been considered to play important role in the regulation of carbohydrate metabolism (Chung et al., 2010). In previous studies, the subfamily II peptides, including gonad/vitellogenesis-inhibiting hormone (GIH or VIH), molt-inhibiting hormone (MIH), and mandibular organ-inhibiting hormone (MOIH), have been reported to regulate the gonad development and molting (Lacombe et al., 1999; Böcking et al., 2002). The inhibitory roles of GIH on ovary development were proposed in *Homarus americanus* (de Kleijn et al., 1994) and proven by silence of GIH expression and injection of GIH recombinant protein (Treerattrakool et al., 2008; Vrinda et al., 2017). In *Metapenaeus ensis*, one type of MIH, MIH-B, showed an inhibitory role in the initiation of vitellogenesis as its expression levels in the eyestalk decreased in the initial phase of ovary maturation (Gu et al., 2002). A recent study revealed that CHH subfamily II members could also regulate male sexual development because silencing of either GIH or MIH could dramatically increase the transcriptional level of *IAG* in *Macrobrachium nipponense* (Li et al., 2015). Except for CHH family hormones, the crustacean female sex hormone in *Scylla paramamosain* was also found to be an inhibitory factor of *IAG* expression (Liu et al., 2018). These findings provide molecular evidence on the “eyestalk-AG-testis” endocrine axis hypothesis. However, most of the key factors upstream the *IAG* in the “eyestalk-AG-testis” endocrine axis, such as hormone receptor, were still poorly understood.

In the present study, two CHH subfamily I peptides (LvCHH1 and LvCHH2) and their putative receptor, a guanylate cyclase (LvGC), were identified as part of the “eyestalk-AG-testis” endocrine axis in the Pacific whiteleg shrimp *L. vannamei*. Tissue distribution analysis showed that both LvCHHs and LvGC displayed sex-biased expression patterns. Functional analysis revealed that both LvCHH1 and LvCHH2 could interact with LvGC and regulate the expression of *LvIAG*.

## Materials and Methods

### Animals and Tissue Collection

Ten adult *L. vannamei*, with a body length of 12.75 ± 0.6 cm, cultured in our lab, were reared in 8 m^3^ fiberglass with air-pumped circulating sea water for 7 days before experiments. The water temperature was monitored regularly and maintained at 26°C. The shrimps were fed with artificial diet twice a day. These shrimps, including five females and five males in the inter-molt stage were used for sampling after 7 days culturing. The molting stages of used shrimp were determined according to the morphology described by Gao et al. (2015). The hemolymph was collected using a syringe preloaded with equal volume of sterilized pre-cooled anticoagulant solution (115 mmol L^–1^ glucose, 27 mmol L^–1^ sodium citrate, 336 mmol L^–1^ NaCl, 9 mmol L^–1^ EDTA⋅Na_2_⋅2H_2_O, pH 7.4). The hemolymph from females and males was mixed as two samples, respectively. The hemocytes were isolated by an immediate centrifugation at 1000 *g* at 4°C for 15 min and preserved in liquid nitrogen. Fourteen tissues, including testis, ovary, gill, stomach, hepatopancreas, lymphoid organ (Oka), heart, intestine, epidermis, ventral nerve cord (VNC), brain, eyestalk (right side), thoracic ganglia (TG), and muscle were dissected. Each tissue from five female and five male shrimps were mixed, respectively. All collected samples were frozen in liquid nitrogen and then stored in −80°C freezer for RNA extraction.

Eyestalk (left side) with the crust removed from five male shrimps were fixed in RNA friendly fixative (RFF) for 48 hours (h) at 4°C. After dehydrating, clearing and embedding, the tissues were sliced into 5 μm for hematoxilin and eosin (H&E) staining and *in situ* hybridization.

### RNA Extraction and cDNA Synthesis

Total RNAs were extracted using RNAiso Plus reagent (TaKaRa, Japan) according to the manufacturer’s instructions. The quality and concentration of total RNAs extracted from different tissues were detected using 1.5% agarose electrophoresis and NanoDrop 2000 (Thermo Fisher Scientific, United States), respectively. The total RNA was pre-treated with DNase to eliminate genomic DNA and the cDNA was synthesized from 1 μg total RNA with PrimeScript RT Reagent Kit (TaKaRa, Japan).

### Gene Cloning and Sequence Analysis

The cDNA sequences of *LvCHH1*, *LvCHH2* and *LvGC*, were obtained from an Illumina-based transcriptome sequencing database of *L. vannamei* constructed in our lab (Wei et al., 2014). Primers *LvCHH1-*F/R, *LvCHH2-*F/R, and LvGC-F/R were designed using Primer Premier 5 software (^1^ other primers were also designed with the software) to amplify the open reading frame (ORF) sequences of *LvCHH1*, *LvCHH2*, and *LvGC*, respectively (Table 1). The PCR was performed using ExTaq (TaKaRa, Japan) as the following procedure: 1 cycle of denaturation at 95°C for 5 min, 40 cycles of denaturation at 94°C for 30 s, annealing at 56°C for 30 s, and extension at 72°C for different times (30 s for *LvCHH1* and *LvCHH2*, and 4 min for *LvGC*), followed by a cycle of extension at 72°C for 10 min. The specific products were assessed by 1.5% agarose gel electrophoresis and purified using OMEGA Gel Extraction Kit (OMEGA, United States). The purified products were cloned into pMD19-T vector (TaKaRa, Japan) and transformed into Trans 5α competent cells. The selected positive clones with expected size of amplified *LvCHH1* and *LvCHH2* products were sent for DNA sequencing.

**TABLE 1:** Primer sequences and corresponding annealing temperature of genes.

**Prime name**	**Primer sequence (5′–3′)**	**Expected size (bp)**	**Annealing temperature (°C)**
LvCHH1-F	AGGACTTCGCTTCAGTCTCG	537	56.5
LvCHH1-R	AGTTTGTCGTCTGGAGGTCG		
LvCHH2-F	GTCCTCGTTCCTGCTGATCT	499	56.5
LvCHH2-R	GGCAACCGAAGGAGAAACTA		
LvCHH1-qF	ACCACAACGAAGTGTTCCTGT	232	56
LvCHH1-qR	CATCCGTGTTTTCAAATCATAGT		
LvCHH2-qF	CTGTTTCCACAACGAGGTATTC	203	56
LvCHH2-qR	GCAACTTAGGATAAAAGGCAAC		
18S-qF	TATACGCTAGTGGAGCTGGAA	136	56
18S-qR	GGGGAGGTAGTGACGAAAAAT		
LvCHH1-dsF	TAATACGACTCACTATAGGGAGGACTTCGCTTCAGTCTCG	577	60
LvCHH1-dsR	TAATACGACTCACTATAGGGAGTTTGTCGTCTGGAGGTCG		
LvCHH2-dsF	TAATACGACTCACTATAGGGGTCCTCGTTCCTGCTGATCT	439	60
LvCHH2-dsR	TAATACGACTCACTATAGGGGGCAACCGAAGGAGAAACTA		
LvCHH1-rpF	GCCATGGCTGATATCGGATCCATGGCCACCTGGGACCGCTC	378	60
LvCHH1-rpR	TTGTCGACGGAGCTCGAATTCTTTGCCGAGCTTCTGCAGGG		
LvCHH2-rpF	GCCATGGCTGATATCGGATCCATGGCTATTACGACTCATGA	363	60
LvCHH2-rpR	TTGTCGACGGAGCTCGAATTCCTTGCCGAGCCTCTGCAGGG		
EGFP-dsF	TAATACGACTCACTATAGGGCAGTGCTTCAGCCGCTACCC	289	60
EGFP-dsR	TAATACGACTCACTATAGGGAGTTCACCTTGATGCCGTTCTT		
LvGC-F	GGAAATGGATGCGCCAATATACT	4667	58
LvGC-R	GTCTATGTACAGCAGTGATATGAG		
LvGC-dsF	TAATACGACTCACTATAGGGAAACTGATGCTGGATAAGATTGG	577	60
LvGC-dsR	TAATACGACTCACTATAGGGCTTCTGAGTGATGGCTGGATTGT		
LvGC-EGFP-F	GAGCTCAAGCTTCGAATTCTGATGAGCCGAAGCCTTGCAG	2841	60
LvGC-EGFP-R	GGTGGCGACCGGTGGATCCCGCCGAGGCCGCTGAACAAA		
LvGC-qF	TCTCCTTCGGCATCATCCTCTAC	86	56
LvGC-qR	GATGTCTTGCACGGTGAACTTAT		
LvCHH1-BD-F	ATGGCCATGGAGGCCGAATTCAAGCGCTCCGGATACTACAAC	308	60
LvCHH1-BD-R	TGCAGGTCGACGGATCCCTATTTGCCGAGCTTCTGCAGGG		
LvCHH2-BD-F	ATGGCCATGGAGGCCGAATTCAAACGCACCACATCGTTCTC	308	60
LvCHH2-BD-R	TGCAGGTCGACGGATCCCTACTTGCCGAGCCTCTGCAGGG		
LvIAG-qF	TTTACTTACATCTCACCGTTATTTCT	188	56
LvIAG-qR	TCTGCTTTCGGATTTCATTG		
LvGC-AD-F	GCCATGGAGGCCAGTGAATTCAAACGCACCACATCGTTCTC	1190	60
LvGC-AD-R	TCGAGCTCGATGGATCCTTAGATGGCCAGCAGGGAATAGG		

The complete ORF regions and deduced amino acid sequences of *LvCHH1*, *LvCHH2*, and *LvGC* were all analyzed using ORF finder^2^. Conserved protein domains were predicted with SMART^3^. Multiple sequences alignments were performed by ClustalW and phylogenic analysis were constructed by the neighbor-joining (NJ) algorithm using the MEGA7^4^. The reliability of the tree was tested by bootstrapping using 1,000 replications.

### PCR Detection on the mRNA Expression of LvCHHs and LvGC

To detect the mRNA expression of *LvCHHs* and *LvGC* in both male and female individuals, primers LvCHH1-qF/qR, *LvCHH2*-qF/qR, and *LvGC*-qF/qR (Table 1) were designed. Semi-quantitative PCR was performed to detect the expression of *LvCHH1* and *LvCHH2* in all tissues following the program: denaturation at 94°C for 5 min; 30 cycles of denaturation at 94°C for 30 s, annealing at 56°C for 30 s and extension at 72°C for 30 s. The PCR products were assessed by electrophoresis on 1.5% agarose gel. The quantitative real-time PCR (qPCR) was performed to detect the expression of *LvCHHs* and *LvGC* on Eppendorf Mastercycler^®^ ep realplex (Eppendorf, Germany) using Toyobo Thunderbird qPCR Mix (Toyobo, Japan). The procedure under the condition described below: denaturation at 94°C for 1 min; 40 cycles of 94°C for 20 s, 56°C for 20 s, and 72°C for 20 s. The specificity of PCR product was checked by melting curve followed the procedure: 95°C for 15 s, 60°C for 15 s, 95°C for 15 s.

### *In situ* Hybridization

#### Transcription of Digoxygenin (DIG)-Labeled Riboprobe

Primers *LvCHH1-*dsF, *LvCHH1-*dsR, *LvCHH2*-dsF, and *LvCHH2*-dsR (Table 1) were designed with a T7 promoter sequence at the 5′ end. The plasmids containing right sequences of *LvCHH1* and *LvCHH2* obtained in Section “Gene Cloning and Sequence Analysis” were extracted using Plasmid Mini Kit I (Omega, United States) and used as the PCR templates for synthesis of sense and antisense RNA probes. The primers *LvCHH1-*dsF/R and *LvCHH1-*F/dsR (Table 1) were used to amplify 577 bp *LvCHH1* fragments, which were used as the templates for synthesis of sense and antisense RNA probe of *LvCHH1*, respectively. The primers *LvCHH2-*dsF/R and *LvCHH2-*F/dsR (Table 1) were used to amplify 439 bp *LvCHH2* fragments, which were as the templates for synthesis of RNA probe of *LvCHH2*, respectively. The PCR program was performed as follows: denaturation at 95°C for 5 min, 40 cycles of 94°C for 30 s, 60°C for 30 s, and 72°C for 30 s, followed by a cycle of extension at 72°C for 10 min. The PCR products were purified using MiniBEST DNA Fragment Purification Kit (TaKaRa, Japan). The purified PCR products were assessed by electrophoresis on 1.5% agarose gel and the concentration was measured by Nanodrop 2000 (Thermo Fisher Scientific, United States). DIG-labeled oligo-nucleotides probes were synthesized using 450 ng DNA templates through *in vitro* transcription using DIG RNA Labeling Mixture (Roche, Germany) and TranscriptAid T7 High Yield Transcription Kit (Thermo Fisher Scientific, United States). After assessing the concentration and quality, the DIG-labeled RNA probes were stored at −80°C for later use.

#### *In situ* Hybridization

Tissues were dehydrated in gradient ethanol and embedded in paraffin. Paraffin-embedded tissues were sectioned into slices of 5 μm, deparaffinized, and hydrated in DEPC treated water. After proteinase K (15 μg/mL) treated at 37°C for 30 min, tissues were washed with PBS for 10 min and fixed in 4% paraformaldehyde at 4°C for 5 min. Then the sections were pre-incubated at 37°C for 3 h in the pre-hybridization buffer (50% formamide deionized, 5 × SSC, 1 μg/μL salmon sperm DNA, 10 × Denhardt’s). Hybridization was performed at 56°C overnight in 500 μL hybridization solution for each sample following general protocol of DIG RNA labeling kit (Roche, Germany). The final concentration of both sense RNA probe and antisense RNA probe were 1 ng μL^–1^. After the hybridization, the slides were washed with 2 × SSC for 2 × 15 min, 1 × SSC for 2 × 15 min, and 0.1 × SSC for 2 × 15 min. The hybridized probes were immunodetected with anti-digoxigenin-AP (1:500 dilution, Roche, Germany) at 4°C for 12 h and visualized by the color reaction using NBT/BCIP Stock Solution (Roche, Germany). The final photos were captured by Nikon Eclipse 80i microscope (Nikon, Japan).

### Subcellular Localization of LvGC Protein in Mammalian 293T Cells

To study the subcellular localization of LvGC, a plasmid containing the predicted signal peptide and transmembrane motif (TM) of LvGC was constructed, designed as pEGFP-LvGC. The restriction endonucleases *Eco*RI and *Bam*HI were used to generate linearized vector of pEGFP-N1. The nucleotide sequences which encoded the predicted signal peptide and TM domains were amplified using primers *LvG*C-EGFP-F/R. In-fusion HD Cloning Kit (Clontech, United States) was used to connect the linearized vector and the PCR product. The plasmid pEGFP-N1 was used as control plasmid. Two micrograms of the plasmids pEGFP-LvGC and pEGFP-N1 were transfected into the mammalian 293T cells with Lipofectamine 3000 Reagent (Thermo Fisher Scientific, United States) following the manufacture’s instruction, respectively. After 48 h culturing, the transfected cells were fixed with 4% paraformaldehyde, stained with 100 ng/ml DAPI (4′, 6-diamidino-2-phenylindole) solution and washed with PBS. The green and blue fluorescence signals were acquired and merged through Nikon Eclipse Ti fluorescence microscope (Nikon, Japan).

### Unilateral Eyestalk Ablation and Detection on the Expression of LvIAG by Real-Time PCR

As *LvCHH1* and *LvCHH2* mainly expressed in male eyestalks (see section “PCR Detection on the mRNA Expression of LvCHHs and LvGC”), the transcripts of *LvIAG* after unilateral eyestalk ablation in adult male *L. vannamei* were detected to study whether LvCHHs could regulate the expression of *LvIAG*. Twenty male shrimp at inter-molt stage, equally divided into experimental group and control group, were temporarily reared in 8 m^3^ fiberglass with air-pumped circulating sea water for 7 days before experiments. The temperature of water was monitored to maintain at 26°C. Unilateral eyestalk of each shrimp in the experimental group was ablated using hot tweezers. No eyestalk was ablated in the individuals from the control group. At 7 days after ablation, AGs were dissected from nine males at inter-molt stage and mixed as three biological replications (*n* = 3, 3, 3) to detect the mRNA expression of *LvIAG*. The total RNAs extraction and cDNA synthesis were performed as described in Section “RNA Extraction and cDNA Synthesis”. SYBR Green-based quantitative real-time PCR (qPCR) was performed using designed primers *LvIAG*-qF/qR and 18S-qF/qR (Table 1). The procedure was running on Eppendorf Mastercycler^®^ ep realplex (Eppendorf, Germany) using SuperReal PreMix Plus (SYBR Green) (Toyobo, Japan) under the process described below: denaturation at 94°C for 1 min; 40 cycles of 94°C for 20 s, 56°C for 20 s, and 72°C for 20 s.

### Preparation of Double-Stranded RNA (dsRNA) and Optimization of dsRNA Dosage

Primers *LvCHH1-*dsF/dsR, *LvCHH2-*dsF/dsR, and *LvGC-*dsF/dsR (Table 1), were designed to amplify cDNA fragments as the templates for dsRNA synthesis of *LvCHH1*, *LvCHH2*, and *LvGC*, respectively. Primers of *EGFP*-dsF/dsR (Table 1) were used to amplify the template DNA fragments of enhanced green fluorescent protein (EGFP) gene for dsRNA synthesis. The PCR was performed as the following procedure: 1 cycle of denaturation at 95°C for 5 min, 40 cycles of denaturation at 94°C for 30 s, annealing at 60°C for 30 s, and extension at 72°C for 30 s, followed by an extension at 72°C for 10 min. The specific products were purified and assessed. The dsRNAs were synthesized with 1 μg template DNA using TranscriptAid T7 High Yield Transcription Kit (Thermo Fisher Scientific, United States). After RNaseA (Thermo Fisher Scientific, United States) digesting, synthesized dsRNAs (dsCHH1, dsCHH2, dsGC, and dsEGFP) were assessed on 1.5% agarose gel, and the concentration was measured by Nanodrop 2000 (Thermo Fisher Scientific, United States) and stored at −80°C until use.

Forty-eight male *L. vannamei*, with a body length of 12.5 ± 0.6 cm and a body weight of 25.4 ± 1.3 g, were chosen to optimize the dosage of dsRNAs, dsCHH1, dsCHH2, dsGC, and dsEGFP. Each group contained four individuals. The efficiency of dsRNAs was detected under different injection dosages, including 1, 2, 4 μg for each shrimp. The same dosage of dsEGFP was injected into control group corresponding to the experiment group. The dsRNA was intramuscularly injected into each individual at the 5^th^ abdominal segment. Eyestalks of four individuals were sampled after 48 h after injection with dsCHH1, dsCHH2, dsGC, and dsEGFP, respectively. Total RNA extraction and cDNA synthesis were the same as described in Section “RNA Extraction and cDNA Synthesis.”

### Detection on the Transcription Level of *LvIAG* and *LvGC* After RNAi

After optimization, 2 μg was chosen for dsCHH1 and dsGC and 4 μg was chosen for dsCHH2 to inject into each shrimp, respectively. The same dosage of dsEGFP was injected into control group corresponding to the experiment group. After 48 h, the AGs were sampled to detect the relative expression of *LvIAG*. At the same time, the cephalothorax and eyestalks were collected to analyze the effects of *LvCHH1, LvCHH2* knockdown on the expression regulation of *LvGC*. Primers (Table 1) used in qPCR were performed as described in Sections “PCR Detection on the mRNA Expression of LvCHHs and LvGC” and “Unilateral Eyestalk Ablation and Detection on the Expression of LvIAG by Real-Time PCR.”

### Expression and Purification of Recombinant Protein

Endonucleases *Eco*RI and *Bam*HI were used to linearize the plasmid pET30a. Primers LvCHH1-rpF/rpR, LvCHH2-rpF/rpR were designed with 15 bp extension homologous of linearized pET30a vector (Novogen, France) ends at both ends to amply the ORF of LvCHH1 and LvCHH2 excluding sequence encoding signal peptide, respectively. The PCR products were purified and inserted into the linearized pET30a using in-fusion cloning reaction with In-fusion HD Cloning Kit (Clontech, United States). The recombinant plasmids, pET30a-CHH1 and pET30a-CHH2, were transformed into *E. coli* BL21 (DE3) competent cell for sequencing.

The recombinant proteins successfully expressed in the inclusion bodies of *E. coli*. rCHH1 and rCHH2 were purified with a gradient urea of 3 and 5M, and dissolved in 8M urea and refolded by gradient dialysis. These recombinant proteins were examined by SDS polyacrylamide gel electrophoresis (SDS-PAGE) and quantified to concentration of 500 ng μL^–1^ by BCA Protein Assay Kit (Tiangen, Beijing) for use.

### Detection on the Transcription Level of LvIAG and LvGC After Recombinant Proteins Injection

Twelve experimental animals were divided into three groups. Each experimental group was injected with 5 μg rCHH1, rCHH2 dissolved in 20 μL PBS, respectively. Control group was injected with 20 μL PBS. These shrimps were reared in fiberglass provided with continuous aeration for 24 h. The temperature of water was maintained at 26°C. After 24 h, AGs, cephalothorax and eyestalks of four individuals were sampled. The Total RNAs extraction and cDNA synthesis were performed following the procedures in Section “RNA Extraction and cDNA Synthesis.” Real time PCR analysis with primers *LvIAG*-qF/qR and *LvGC*-qF/qR (Table 1) were performed as described in Section “Unilateral Eyestalk Ablation and Detection on the Expression of LvIAG by Real-Time PCR.”

### Yeast Two-Hybrid Assay

To further study the relation between the LvCHHs and LvGC, yeast two-hybrid system was performed. Plasmids, pGADT7 and pGBKT7 (TaKaRa, Japan), were digested with restriction endonucleases *Eco*RI and *Bam*HI to generate linearized vectors. The nucleotide sequences encoding the mature peptide of LvCHHs and the extracellular domain of LvGC were amplified using primers *LvCHH1*-BD-F/R, *LvCHH2*-BD-F/R, and *LvGC*-AD-F/R (Table 1). The expression vectors were constructed as described in Section “Subcellular Localization of LvGC Protein in Mammalian 293T Cells.” The plasmids combinations LvCHH1 with LvGC, LvCHH2 with LvGC were co-transformed into yeast strain Y2H Gold by the lithium acetate transformation procedure following the Matchmaker protocol manual (Clontech, United States). The plasmids combination, pGBK-p53 with pGAD-T-antigen, was used for positive control and the combination pGBK-Lam and pGAD-T-antigen was used for negative control. After culturing on SD/-Leu/-Trp (DDO) plates, growing 3–5 days at 30°C, all clones growing were collected and coated on SD/-Leu/-Trp/-His/-Ade/X-α-gal/Aba (QDO/X/A) plates to perform β-Galactosidase activity analysis.

### Data Analysis

In all qPCR experiments, each sample was set four technical replications. The relative expression level of *LvCHH1*, *LvCHH2*, *LvGC*, and *LvIAG* were all calculated using the comparative Ct method with the formula 2^–ΔΔCt^. Statistical analysis used unpaired two tailed *t*-test and Tukey multiple comparison test and performed by GraphPad Prism software (version 5.0). The *P*-value less than 0.05 was considered statistically significant.

## Results

### LvCHH1 and LvCHH2 Are CHH Subfamily I Members

**FIGURE 1:**
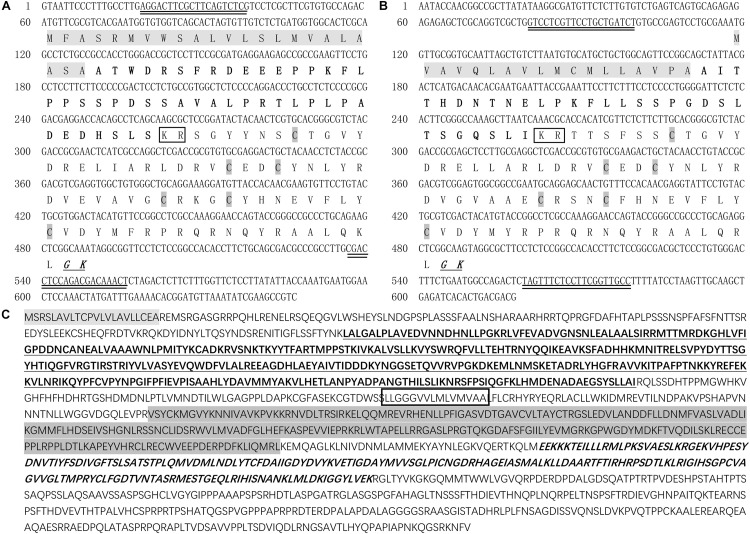
Deduced amino acid sequences and predicted domains of LvCHH1, LvCHH2, and LvGC. **(A)** Deduced amino acid sequence and predicted domains of LvCHH1. **(B)** Deduced amino acid sequence and predicted domains of LvCHH2. Signal peptide was showed with light gray box. The CHH-precursor-related peptide was bolded. Dibasic residue cleavage signal (KR) was marked in the box. Amidation signal (GK) was bolded, italic and underline. The conversed cysteine residues were shown as italic with dark gray box. **(C)** Deduced amino acid sequence and predicted domains of LvGC. Signal peptide was showed in light gray. The receptor domain was bolded and underlined. The transmembrane domain marked in the box. The protein kinase-like domain was showed in dark gray. The guanylate cyclase catalytic domain was bolded and italic. The primers used in RNAi were double underlined.

The ORF of *LvCHH1* (Accession number: MK732901) was 432 bp in length, encoding 143 amino acid residues, and the ORF of *LvCHH2* (Accession number: MK732902) was 375 bp in length, encoding 124 amino acid residues. Both LvCHH1 and LvCHH2 belonged to CHH subfamily I. LvCHH1 is composed of a 23 aa signal peptide, a 44 aa CHH-precursor-related peptide (CPRP) and a 76 aa mature protein (Figure 1A). LvCHH2 is composed of an 18 aa signal peptide, a 30 aa CPRP, and a 76 aa mature protein (Figure 1B). A processing signal KR and an amidation signal GK were found in both LvCHH1 and LvCHH2.

**FIGURE 2:**
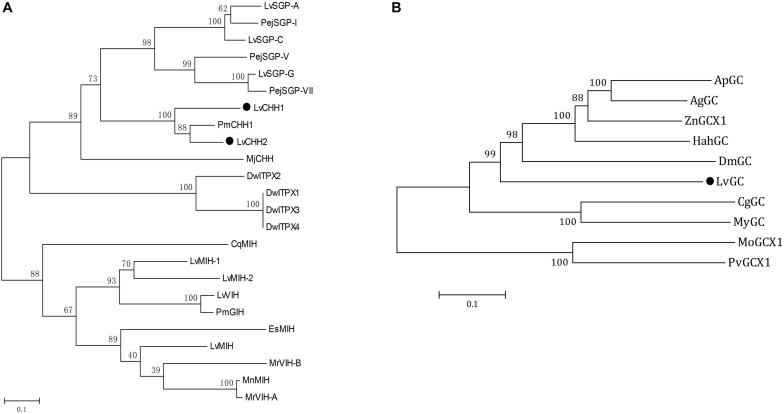
Phylogenetic analyses of CHHs and GCs. **(A)** Phylogenetic analysis of LvCHH1 and LvCHH2 (solid circle) from *Litopenaeus vannamei* and homologous proteins from other species (*Drosophila willistoni*, DwITPX1/XP_015034465, DwITPX2/XP_015034467, DwITPX3/XP_002062858, DwITPX4/XP_023037604; *Penaeus japonicus*, PejSGP-I/BAA22560, PejSGP-V/BAA22561, PejSGP-VII/BAA22562; *Marsupenaeus japonicus*, MjCHH/BAE78493; *P. monodon*, PmCHH1/AAQ24525, PmGIH/AMQ67879; *C. quadricarinatus*, CqMIH/ACX55057; *Eriocheir sinensis*, EsMIH/ABC68517; *M. nipponense*, MnMIH/AIP90070; *M. rosenbergii*, MrVIH-A/AAL37948, MrVIH-B/AAL37949; *L. vannamei*, LvSGP-A/BBA57870.1, LvSGP-C/BBA57874.1 LvSGP-G/BBA57873, LvMIH/ATN45407, LvMIH-1/ABD73291, LvMIH-2/ABD73292, LvVIH/AGX26044). **(B)** Phylogenetic analysis of LvGC (solid circle) from *L. vannamei* and homologous proteins from other species (*Agrilus planipennis*, ApGC/XP_025832416; *Anoplophora glabripennis*, AgGC/XP_023312344; *Zootermopsis nevadensis*, ZnX1/XP_021915041; *Halyomorpha halys*, HahGC/XP_014284869; *Daphnia magna*, DmGC/KZS20192; *Crassostrea gigas*, CgGC/EKC28433; *Mizuhopecten yessoensis*, MyGC/OWF46460; *Microtus ochrogaster*, MoGCX1/XP_005362150; *Pogona vitticeps*, PvGCX1/XP_020666428). Bootstraps were performed with 1000 replicates to ensure reliability.

Phylogenetic analysis showed that LvCHH2 were first clustered with CHH from *Penaeus monodon* and then clustered with LvCHH1. They were all clustered together with other type I CHHs from *L. vannamei* and *Marsupenaeus japonicus*. In addition, the neuropeptides from CHH subfamily II were all clustered into another branch (Figure 2A).

### LvGC Is a Membrane-Bound Guanylate Cyclase

Several partial transcripts encoding LvGC were obtained from the transcriptome above-mentioned (Wei et al., 2014). The whole transcript of LvGC was gained after PCR amplification and sequencing. The ORF of *LvGC* was 4,551 bp (Accession number: MK732903) encoding 1,516 aa residues (Figure 1C). The deduced amino acid sequence of LvGC contained the conserved domains of guanylate cyclase family members, including signal peptide (Met^1^-Ala^22^), a ligand binding domain (Leu^167^-Ile^536^), a transmembrane domain (Leu^607^- Leu^622^), a protein kinase-like domain (Val^681^- Leu^929^) and a guanylate cyclase catalytic domain (Glu^968^-Lys^1162^). Phylogenetic analysis showed that LvGC was first clustered together with GC from *Limulus polyphemus*, and then with members from insects (Figure 2B).

**FIGURE 3:**
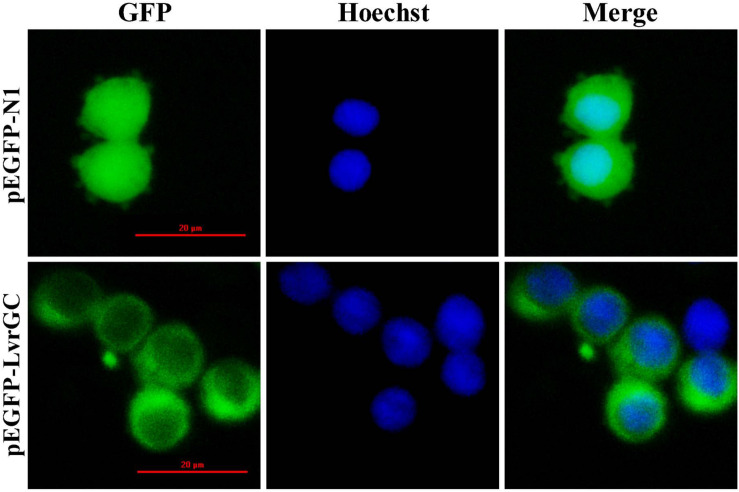
Location of LvGC in HEK293. GFP showed the observation excited by blue light. Hoechst showed the observation excited by green light. Merge showed the merged observation excited by blue light and green light.

The predicted sequence encoding the transmembrane region of LvGC was sub-cloned into the plasmid pEGFP-N1 and transfected into 293T cells. Subcellular localization analysis revealed that the fluorescence signals expressed by constructed plasmid pEGFP-LvGC were mainly detected on the cell membrane of 293T cells, while the fluorescence signals expressed by the control plasmid pEGFP-N1 were in the cytoplasm of 293T cells (Figure 3). The result confirms that LvGC is a membrane-bound guanylyl cyclase.

### Both LvCHHs and LvGC Show Sex-Biased Expression Patterns

**FIGURE 4:**
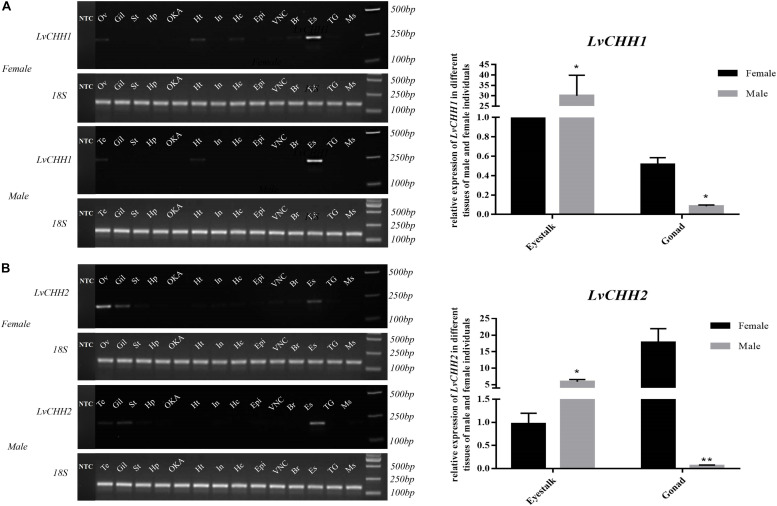
Distribution analysis of *LvCHH1* **(A)** and *LvCHH2* **(B)** in different tissues of male and female *L. vannamei* by RT-PCR and qPCR. 18S rRNA gene was used as the internal reference. Ov, ovary; Te, testis, Gil, gill; St, stomach; Hp, hepatopancreas; OKA, lymph organ; Ht, heart; In, intestine; Hc, hemocyte; Epi, epidermis; VNC, ventral nerve cord; Br, brain; Es, eyestalk; TG, thoracic ganglia; Ms, muscle; NTC, no-template controls (which were detected separately on another gel). Expression levels of target genes were detected in three replicates for each tissue. Significant differences of the gene expression levels were marked with one (*P* < 0.05) or two (*P* < 0.001) stars.

**FIGURE 5:**
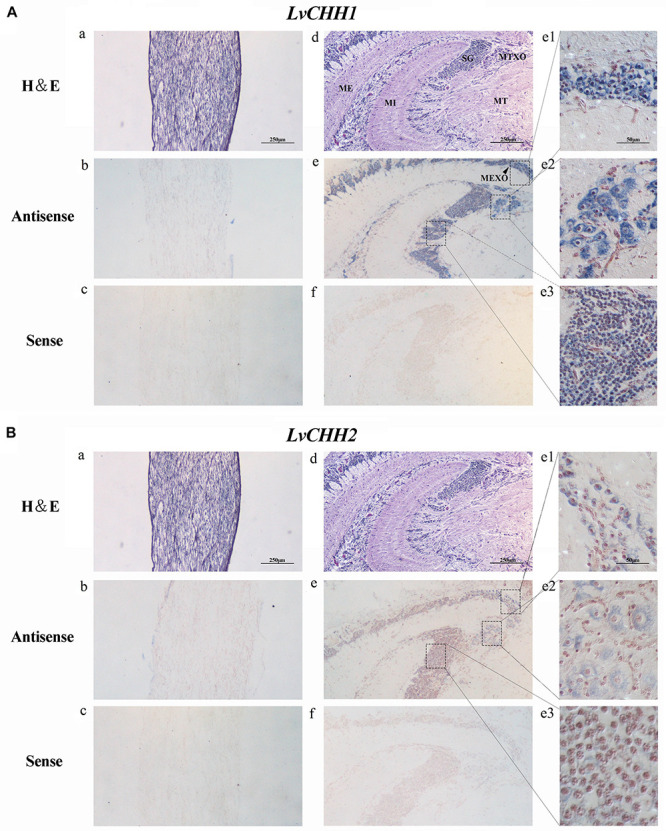
Localization of *LvCHH1* **(A)** and *LvCHH2* **(B)** transcripts in the eyestalk of *L. vannamei*. Hematoxylin–Eosin (H&E) staining **(a,d)** and sense probe **(c,f)** were used as controls of the antisense probe hybridization **(b,e)**. Partial picture was enlarged into **e1** (MEXO), **e2** (MTXO), and **e3** (SG). MTXO, medulla terminalis X-organ; MEXO, medulla externa X-organ; SG, sinus gland; MI, medulla interna; ME, medulla externa; MT, medulla terminalis.

**FIGURE 6:**
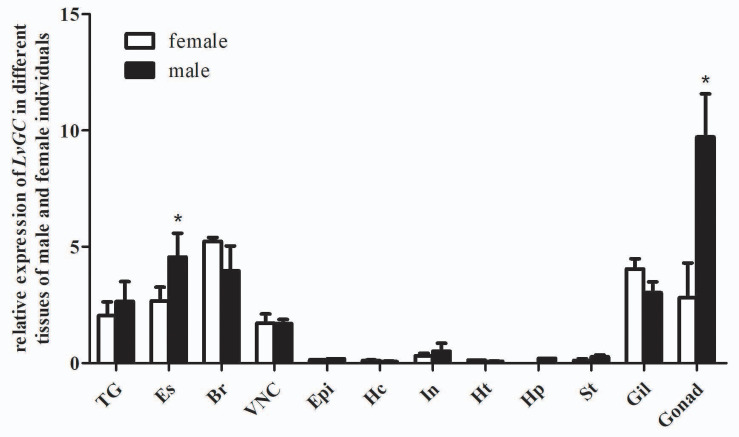
Distribution of *LvGC* in different tissues of male and female *L. vannamei*. 18S rRNA gene was used as the internal reference. TG, thoracic ganglia; Es, eyestalk; Br, brain; VNC, ventral nerve cord; Epi, epidermis; Hc, hemocyte; In, intestine; Ht, heart; Hp, hepatopancreas; St, stomach; Gil, gill; Ov, ovary; Te, testis. Expression levels of target genes were detected in three replicates for each tissue. Significant differences (*P* < 0.05) of *LvGC* expression in male and female tissues were shown with stars (^∗^).

Tissue distribution and *in situ* hybridization analyses of *LvCHHs* and *LvGC* were performed to detect whether they were involved in sexual development. Both *LvCHH1* and *LvCHH2* transcripts were mainly detected in the eyestalk. The transcripts of both *LvCHH1* and *LvCHH2* were apparently higher in male eyestalks than those in female ones (Figure 4). Meanwhile, *LvCHH2* was apparently detected in ovary and was higher thant that in testis (Figure 4B). *In situ* hybridization analysis of eyestalk showed that the transcripts of both *LvCHH1* (Figure 5A) and *LvCHH2* (Figure 5B) were located in the secretory cells of the medulla terminalis X-organ (MTXO), medulla externa X-organ (MEXO) and sinus gland (SG). The expression profile of *LvGC* was shown in Figure 6. The transcripts of *LvGC* were mainly detected in gonads, eyestalk, gill and nerve system in both male and female individuals. However, there was no expression of *LvGC* in AG tissue (with a Ct value more than 36 in the qPCR analysis results). In gonads and eyestalk, *LvGC* showed significantly higher expression level in males than that in females. These data revealed that both *LvCHHs* and *LvGC* showed sex-biased expression patterns, indicating their involvement in sexual development.

### LvCHH1 and LvCHH2 Are Inhibitory Factors of LvIAG Expression

**FIGURE 7:**
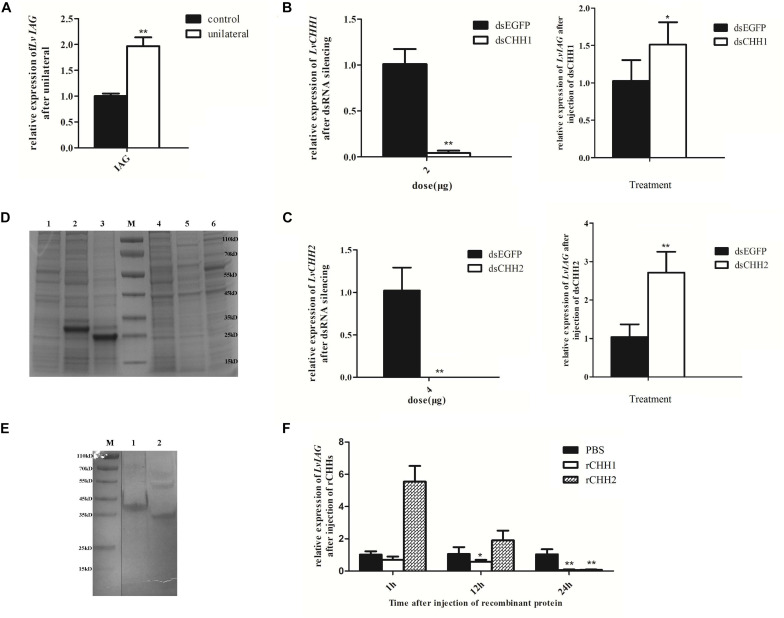
The inhibitory effects of LvCHH1 and LvCHH2 on the expression of *LvIAG*. **(A)** Expression level of *LvIAG* after unilateral eyestalk ablation. **(B)** Expression levels of *LvIAG* after *LvCHH1* knockdown. **(C)** Expression levels of *LvIAG* after *LvCHH2* knockdown. dsEGFP, injected with dsEGFP; dsCHH1, injected with dsCHH1; dsCHH2, injected with dsCHH2. **(D)** The recombination proteins of LvCHHs. 1–3, inclusion body; 1, pET30a; 2, rCHH1; 3, rCHH2; 4–6, supernatant; 4, pET30a; 5, rCHH1; 6, rCHH2; M, protein ladder. **(E)** The recombination proteins of LvCHHs after purification and refolding. 1, rCHH1; 2, rCHH2; M, protein ladder. **(F)** Expression levels of *LvIAG* after injection with rCHH1 and rCHH2. PBS, injected with PBS; rCHH1, injected with rCHH1; rCHH2, injected with rCHH2. Expression levels of target genes were detected in three replicates. Significant differences of the gene expression levels between two treatments were shown with a star (^∗^) at *P* < 0.05 or two stars (^∗∗^) at *P* < 0.01.

After 7 days unilateral eyestalk ablation, the expression level of *LvIAG* was significantly increased by 180% when compared to that in the control shrimp (Figure 7A). DsRNA-mediated RNA interference of *LvCHH1* and *LvCHH2* achieved similar results. After knockdown the expression level of *LvCHH1* was reduced by 96% while the expression level of *LvIAG* increased by 147.6% when compared to that in control shrimp (Figure 7B). After knockdown the expression level of *LvCHH2* was reduced by 99% while the expression level of *LvIAG* increased by 260% when compared to that in control shrimp (Figure 7C). The results showed that knockdown of either *LvCHH1* or *LvCHH2* increased the expression of *LvIAG*.

Recombinant LvCHH1 (rCHH1) protein or LvCHH2 (rCHH2) protein was injected into shrimp to study their effects on inhibiting *LvIAG* expression. The *E. coli* expressed rCHH1 and rCHH2 were in inclusion bodies with distinct bands on SDS-PAGE at weight of 30 and 25 kDa, which were consisted with the predicted molecular weight of them (Figure 7D). The rCHH1 and rCHH2 proteins in inclusion bodies were dissolved and refolded with a gradient of urea solutions and then obtained the purified rCHH1 and rCHH2 proteins (Figure 7E). After injecting rCHH1 or rCHH2 proteins for 24 h, the expression levels of *LvIAG* were significantly decrease by 94 and 92%, respectively (Figure 7F). The results showed that injection of either recombinant LvCHH1 or LvCHH2 could inhibit the expression of *LvIAG*.

### LvGC Is a Putative Receptor of LvCHH1 and LvCHH2 and Inhibits LvIAG Expression

**FIGURE 8:**
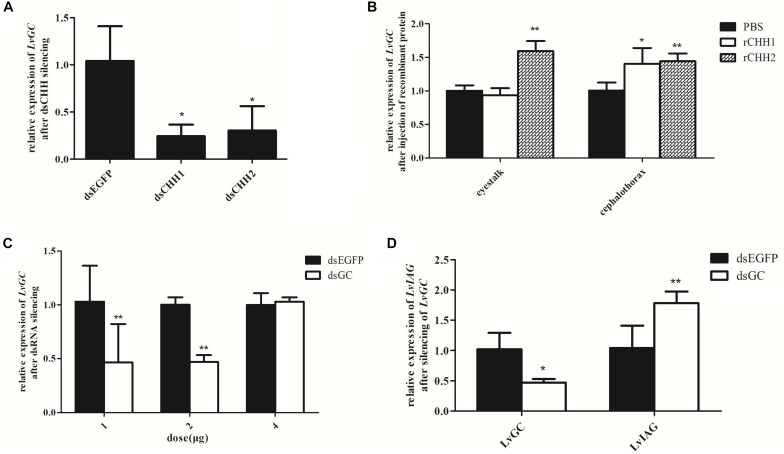
Expression relationships among LvGC, LvCHHs, and LvIAG. Expression levels of *LvGC* after LvCHHs knockdown or recombinant protein injection. **(A)** The expression level of *LvGC* after *LvCHHs* knockdown. dsEGFP, injected with dsEGFP; dsCHH1, injected with dsCHH1; dsCHH2, injected with dsCHH2. **(B)** The expression level of *LvGC* after injection with rCHHs. **(C)** Silencing efficiency on *LvGC* using different dosage of dsRNA. **(D)** Expression changes of *LvIAG* after *LvGC* knockdown. PBS, injected with PBS; rCHH1, injected with rCHH1; rCHH2, injected with rCHH2; dsEGFP, injected with dsEGFP; dsGC, injected with dsGC. Expression levels of target genes were detected in three replicates. Significant differences of the gene expression levels between two treatments were shown with a star (^∗^) at *P* < 0.05 or two stars (^∗∗^) at *P* < 0.01.

As LvGC was found to be a membrane-bound guanylyl cyclase and showed similar sex-biased expression patterns with LvCHH1 and LvCHH2, further studies were performed to detect whether LvGC was the receptor of LvCHH1 and LvCHH2. Firstly, the transcriptional level of *LvGC* was detected after *LvCHH1* or *LvCHH2* silencing. When the expression levels of *LvCHH1* and *LvCHH2* were knocked-down by 96% (Figure 7B, left) and 99% (Figure 7C, left), the transcriptional levels of *LvGC* were down-regulated by 24.2 and 30.3%, respectively (Figure 8A). Then, the transcriptional level of *LvGC* was detected after rCHHs injection. As shown in Figure 8B, the transcriptional level of *LvGC* in cephalothorax was up-regulated by about 40% while no obvious change in eyestalk after injection of rCHH1. After injection of rCHH2, the expression levels of *LvGC* were significantly increased by about 60 and 44% in eyestalk and cephalothorax, respectively.

**FIGURE 9:**
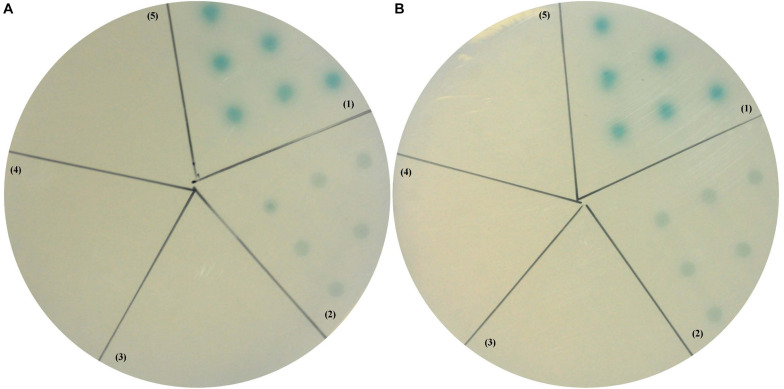
Yeast two-hybrid assay between LvGC and LvCHH1 **(A)** or LvCHH2 **(B)**. (1) A-B, pGBK-p53 and pGAD-T-antigen for positive control; (2) A, pGAD-LvGC and pGBK-LvCHH1 plasmids; B, pGAD-LvGC and pGBK-LvCHH2 plasmids; (3) A, pGAD-T7 and pGBK-LvCHH1 plasmids; B, pGAD-T7 and pGBK-LvCHH2 plasmids; (4) A-B, pGAD-LvGC and pGBK-T7 plasmids; (5) A-B, pGBK-Lam and pGAD-T-antigen for negative control.

Yeast two-hybrid assay was then performed to further investigate whether there were interactions between LvCHHs and LvGC. When the positive plasmids combination pGBK-p53 and pGAD-T antigen were co-transformed into yeast cells, the reporter gene was activated and the colonies turned blue (Figures 9A,B, zone 1). When pGBK-CHH1 and pGAD-LvGC plasmids or pGBK-CHH2 and pGAD-LvGC were co-expressed in yeast cells, the colonies turned lighter blue (Figures 9A,B, zone 2). In self-activation and negative controls, the reporter gene was not activated and no blue signal was detected (Figures 9A,B, zones 3–5).

In order to know whether *LvGC* could also influence the expression of *LvIAG*, the expression level of *LvIAG* was detected after silencing of *LvGC*. When the expression level of *LvGC* was knocked-down by 53% (Figure 8C), the transcriptional level of *LvIAG* was up-regulated by 78% (Figure 8D). These results suggested that LvGC was the putative receptor of LvCHH1 and LvCHH2, and it could also show inhibitory effect, like LvCHH1 and LvCHH2, on the expression of *LvIAG*.

## Discussion

In crustacean, the “eyestalk-AG-testis” endocrine axis has been suggested to regulate male sexual differentiation (Khalaila et al., 2002). As the functional core of the axis, AG controls male sexual differentiation through secreting the sexual hormone IAG (Ventura and Sagi, 2012). We previously identified a male specific expressed IAG receptor, FcIAGR, from the shrimp *F. chinensis* (Guo et al., 2018). Knockdown of FcIAGR expression in a late developmental stage of shrimp retarded testis development, which provided evidence to the downstream of the “eyestalk-AG-testis” endocrine axis. However, the upstream regulatory mechanism of the axis is still lack of reliable evidence.

The X-organ/sinus-gland (XO-SG) complex in the eyestalk is the major neuroendocrine system in crustacean suggested to be an upstream regulator in various endocrine axes. Sexual development related function of neurohormones mainly referred to eyestalk expressed CHH subfamily II members, which were considered as inhibitory factors on ovary development (de Kleijn et al., 1994; Gu et al., 2002; Treerattrakool et al., 2008; Vrinda et al., 2017) and IAG expression (Li et al., 2015). On the other hand, CHH subfamily I members were usually deemed as hyperglycemic factors or osmo-regulators for their functions in regulating crustacean hemolymph glucose levels and ion transport (Chung et al., 2010). In *L. vannamei*, a striking expansion of CHH family genes was found in recent study and three major clades were identified in type I CHH peptides (Zhang et al., 2019). The type Ic clade peptides, which was penaeid-specific and had significant expansion in the genome, could regulate molting, reproduction, energetics, and ionic metabolism (Montagné et al., 2010; Zhang et al., 2019). This suggests that CHHs in *L. vannamei* diverse in expression patterns (such as co-expression and co-functionality) during regulating the same physiological process, and might also participate in other less-well-studied physiological metabolism processes. CHH family genes were initially considered specifically expressed in eyestalk but were also detected in gut, pericardial organ and sub-esophageal ganglion in subsequent researches (Chang et al., 1999; Chung et al., 1999; Webster et al., 2000; Dircksen et al., 2001). The present LvCHH1 and LvCHH2 belong to CHH subfamily I members because they have the conversed CPRP domain, the processing signal KR and the amidation signal GK (Chan et al., 2003). They show sex-biased expression patterns both in eyestalks and gonads. The high expression levels of *LvCHH1* and *LvCHH2* in male eyestalk rather than in female eyestalk indicated their involvement in male sexual development in shrimp. The higher level of LvCHH1 and LvCHH2 transcripts in ovary than in testis indicated that the two LvCHHs might also function in female reproduction.

As part of the “eyestalk-AG-testis” endocrine axis, the XO-SG complex was found to have an inhibitory effect on AG development or *IAG* expression. Eyestalk ablation in decapod crustaceans caused hypertrophy of the AG (Khalaila et al., 2002) and up-regulation of *IAG* transcripts (Chung et al., 2011; Li et al., 2015). The expression level of *LvIAG* was significantly up-regulated after unilateral eyestalk ablation. It indicates that the eyestalk expresses inhibitory factors that regulate *LvIAG* expression. Similar increase in *LvIAG* expression was achieved through knockdown of *LvCHH1* and *LvCHH2*, which was similar with the results in *M. nipponense* after silencing of VIH and MIH (Li et al., 2015). In further, injection of recombinant LvCHH1 and LvCHH2 protein inhibited the expression level of *LvIAG*. Collectively these results suggest that LvCHH1 and LvCHH2, which exhibit sex biased expression patterns in male and female eyestalks, are the inhibitory factors in shrimp eyestalks that play negatively regulatory activities on *LvIAG* expression.

In lobster, membrane guanylate cyclase was proposed to be the receptor of CHH because CHH could elevate cyclic GMP (cGMP) levels and had no effect on soluble guanylate cyclase (Goy, 1990). This was further supported by evidence from *Carcinus maenas*, in which CHH could also increase cGMP levels (Chung and Webster, 2006). Sequence analysis and subcellular localization assay demonstrated that LvGC was a membrane guanylate cyclase. Gene knockdown, recombinant protein injection and yeast two-hybrid assay suggested unanimously that LvGC was the putative receptor for LvCHH1 and LvCHH2. Furthermore, LvGC also showed a sex-biased expression pattern in eyestalk which affected *LvIAG* expression as well. These data indicated that LvGC had similar biological function to that of LvCHH1 and LvCHH2 on male sexual development in shrimp. Taken all these data together, LvCHH1, LvCHH2, and LvGC are suggested to be important upstream components in shrimp “eyestalk-AG-testis” endocrine axis. However, no transcript of LvGC was detected in AG, indicating that LvCHH might indirectly regulate the expression of *LvIAG*.

## Conclusion

The present study characterized two CHH genes, *LvCHH1* and *LvCHH2*, and their putative receptor gene *LvGC* from *L. vannamei*. *LvCHH1* and *LvCHH2* encode CHH subfamily I members and LvGC encodes a membrane-bound guanylate cyclase. They all show sex-biased expression patterns, in which they have higher expression level in male eyestalk than in female eyestalk, while they exhibit higher expression level in ovary than in testis. RNAi, recombinant protein injection and yeast two-hybrid experiments show that LvGC is the receptor for LvCHH1 and LvCHH2. They all could inhibit the expression of LvIAG, suggesting that they are important regulators in male sexual development in shrimp. The present data provide new evidence to support the hypothesis that the “eyestalk-AG-testis” endocrine axis regulates male sexual development in crustaceans.

## Data Availability Statement

The datasets generated for this study can be found in the NCBI Bankit accession numbers: MK732901, MK732902, and MK732903.

## Author Contributions

SL and FL designed the research. QG, SL, and XL performed the research. QG and SL analyzed the data. QG, SL, JX, RM, AS, and FL wrote the manuscript. All authors reviewed the manuscript.

## Conflict of Interest

The authors declare that the research was conducted in the absence of any commercial or financial relationships that could be construed as a potential conflict of interest.

## Footnotes

1 www.premierbiosoft.com/primerdesign/

2 https://www.ncbi.nlm.nih.gov/orffinder/

3 http://smart.embl-heidelberg.de/

4 https://megasoftware.net/
