# Corrigendum: Sex-Biased CHHs and Their Putative Receptor Regulate the Expression of IAG Gene in the Shrimp *Litopenaeus vannamei*

**DOI:** 10.3389/fphys.2021.705293

**Published:** 2021-05-31

**Authors:** Qing Guo, Shihao Li, Xinjia Lv, Jianhai Xiang, Rivka Manor, Amir Sagi, Fuhua Li

**Affiliations:** ^1^Key Laboratory of Experimental Marine Biology, Institute of Oceanology, Chinese Academy of Sciences, Qingdao, China; ^2^Laboratory for Marine Biology and Biotechnology, Qingdao National Laboratory for Marine Science and Technology, Qingdao, China; ^3^Center for Ocean Mega-Science, Chinese Academy of Sciences, Qingdao, China; ^4^University of Chinese Academy of Sciences, Beijing, China; ^5^Department of Life Sciences and the National Institute for Biotechnology in the Negev, Ben-Gurion University of the Negev, Beersheba, Israel

**Keywords:** CHH, “eyestalk-AG-testis” endocrine axis, guanylate cyclase, IAG, male sexual differentiation

There is an error in the Funding statement. The correct number for 2018YFD0900502 is 2018YFD0900202. The correct Funding statement is below:

This work was financially supported by the National Key Research and Development Program of China (2018YFD0900202), the Joint NSFC-ISF Research Program (31861143047), the China Agriculture Research System-48 (CARS-48), and the ISF-NSFC Joint Research Program (Grant No. 2368/18).

The authors apologize for this error and state that this does not change the scientific conclusions of the article in any way. The original article has been updated.

